# Hybrid capture vs. PCR screening of cervical human papilloma virus infections. Cytological and histological associations in 1270 women

**DOI:** 10.1186/1471-2407-10-53

**Published:** 2010-02-22

**Authors:** Sotirios Tsiodras, John Georgoulakis, Aikaterini Chranioti, Zanis Voulgaris, Amanda Psyrri, Angeliki Tsivilika, John Panayiotides, Petros Karakitsos

**Affiliations:** 1 4th Dept of Internal Medicine, Attikon University Hospital, University of Athens Med. Sch. Athens, Greece; 2 Department of Cytopathology, Attikon University Hospital, University of Athens Med. Sch. Athens, Greece; 3 Department of Histology and Embryology, University of Athens Med. Sch. Athens, Greece; 4 1st Department of Obstetrics and Gynecology, University of Athens Med. Sch. Athens, Greece; 5 Department of Internal Medicine and Oncology, University of Athens Med. Sch. Athens, Greece; 6 Department of Cytopathology, Alexandra Hospital, Athens, Greece; 7 2nd Department of Pathology, Attikon University Hospital, Athens University Medical School, Athens, Greece

## Abstract

**Background:**

We evaluated two molecular methods of HPV detection and their correlation with cytological and histological diagnosis in a large sample of Greek women.

**Methods:**

All women with liquid-based cytology performed at a University Hospital between 2000 and 2003 were included. The Hybrid Capture 2 (HC2) kit and in house Polymerase Chain Reaction (PCR) were used for HPV DNA detection. Cervical biopsy was performed for women with ASCUS+ cytology, HPV detection, or abnormal colposcopy. Positive (PLR) and negative (NLR) likelihood ratios were calculated for cytology and HPV molecular testing for the prediction of CIN2 and greater histology.

**Results:**

Of the 1270 women evaluated 241 (18.5%) had abnormal cytology. Cytology diagnosed high-grade squamous intraepithelial lesion (HSIL) or invasive carcinoma in 21(1.7%) cases whereas 26 (2%) women had CIN2+ or greater histology. PCR detected HPV in 397/1270 (31.3%) and HC2 in 260/1270 (20.4%) samples. Both molecular tests exhibited high reproducibility (Cohen's kappa value 0.691, 95% CI: 0.664 - 0.718). Positive likelihood ratios (PLR) of 9.4, 3.8 and 3.4 and negative likelihood ratios of 0.13, 0.21, and 0 were noted for ≥ LSIL, any positive HC2 or any positive PCR-HPV testing, for predicting CIN2+ histology, respectively. All CIN 3+ lesions harbored high risk oncogenic HPV type infections.

**Conclusions:**

HPV infection was found in a large proportion of this population and was associated with CIN 2/3 lesions and infiltrating carcinomas. Thin prep testing and HPV detection by HC2 or PCR performed very well with regards to identifying high grade lesions in an environment with experienced examiners.

## Background

Large epidemiological studies performed over the last two decades have identified infection with human papilloma virus (HPV) as a necessary cause for cervical cancer[1,2]. Furthermore, newer molecular techniques have greatly helped in identifying the association of high-risk HPV types with the development of precancerous lesions, as well as their role in cervical carcinogenesis[3,4]. Since the vast majority of invasive cervical carcinoma cases are associated with HPV type 16 or 18 infections[5,6], efforts have been made to develop preventive measures for infections with these high risk HPV types: two specific vaccines targeting infection with HPV types 16 and 18 have recently been introduced[7].

Specific HPV testing may not be appropriate as a primary screening due to claims that it lacks specificity[8] but it may assist in the management of women with cytological changes[9]. The association of findings from conventional cytological testing with those of newer molecular techniques is of great importance and helps to better understand the evolution of HPV infection in different epidemiological settings.

The purpose of the current study is to evaluate cytological findings from a large observational population sample in association with identification of HPV infection using newer molecular techniques, such as Hybrid Capture 2 and PCR. Furthermore, we correlate these findings with histological diagnosis.

## Methods

This is a cross-sectional study performed at all specimens sent for diagnostic cytology at the Departments of Histology and Embryology, and the Department of Cytopathology of the National and Capodistrian University of Athens Medical School. Samples are addressed to these departments every day from many community and hospital-based Gynecology clinics covering the metropolitan area of Athens (roughly a population of 3.8 million people). Informed consent was obtained from all women included in this study that was performed between January 2000 and December 2003.

### Cytological diagnosis

Samples for the ThinPrep Pap test were collected by means of a Brun's-like brush. The PreservCyt^® ^vials (Cytyc Inc, Boxborough, MA, USA) containing the cell samples were addressed to the aforementioned Departments for preparation of thin-layer slides using the ThinPrep 2000 Automated Slide Processor^® ^(Cytyc, Boxborough, MA, USA) according to the manufacturer' instructions. Cytological findings were interpreted according to the Bethesda classification system [10] and were classified as follows a) within normal limits; b) atypical squamous cells of undetermined significance (ASC-US); c) low-grade squamous intraepithelial lesion (LSIL); d) high-grade squamous intraepithelial lesion (HSIL); e) squamous cell carcinoma (SCC) or adeno-carcinoma. An experienced cytopathologist whose diagnostic experience exceeds 10 years examined all samples.

### Histological diagnosis

A cervical biopsy was performed if ThinPrep testing revealed ASCUS and above cytological categories or there was positive HPV testing or there was a visible lesion upon colposcopy. Biopsy was performed by an experienced colposcopist (in practice for more than 10 years) as part of the study protocol. All women with indications consented to this procedure. The research was performed with the approval of the Institutional Review Board and in compliance with the Helsinki declaration. The three-tiered cervical intraepithelial neoplasia (CIN) grading system was used for histological diagnosis [11]. All cases were diagnosed by an experienced pathologist (more than 10 years experience). In case histology showed a CIN 2 or CIN 3 or invasive carcinoma the patient was referred for appropriate treatment.

### HPV DNA detection

Two techniques were used simultaneously:

a) The commercially available Hybrid Capture 2 (HC2) kit that was used according to the manufacturer's instructions (Digene Corporation, Gaithersburg, MD). Briefly, HC2 is a signal amplification assay that uses a combination of antibody capture and chemiluminescent signal detection. An RNA probe cocktail (full genome probes) that detects 13 high-risk HPV types (16/18/31/33/35/39/45/51/52/56/58/59/68) and 5 low-risk (6/11/42/43/44) HPV types is used in the reaction with the target DNA. The RNA:DNA hybrids are captured onto a solid phase coated with universal capture antibodies specific for the RNA:DNA hybrids. Capture RNA:DNA hybrids are detected with multiple antibodies conjugated to alkaline phoshatase. The signal resulting from the chemiluminescent reaction is read and the results are interpreted. For each specimen relative light unit/cutoff values were calculated as the ratio of the specimen luminescence relative to the luminescence of the 1.0 pg/ml HPV-16 cut-off standard and reflect a semi-quantitative value of the cumulative viral burden from one or more of the examined genotypes. A relative light unit/cutoff value of ≥ 1 was considered as a positive result. Specimens testing positive for high-risk HPV types or low risk HPV types only were classified as HPV-high risk or HPV-low risk respectively, whereas specimens testing positive for both low and high risk HPV types were classified as HPV low-high risk.

b) An in house Polymerase Chain Reaction (PCR) to detect HPV DNA presence. DNA was extracted and purified from 3 ml of residual ThinPrep samples using a commercially available kit according to the manufacturer's instructions (QIAamp DNA mini kit, Tissue protocol, QIAGEN Inc, Valencia, Ca). The quantity and integrity of the DNA extracts was monitored through spectrophotometer readings and amplification of β-globin gene (endogenous control). The extracted DNA was tested for the presence of HPV DNA by PCR using the consensus (general) primers GP5+/GP6+ following a previously published protocol[12]. These primers amplify a fragment of approximately 150 base pairs (bp) from the L1 region of 22 anogenital HPV genotypes (types 6, 11, 13, 16, 18, 30-33, 35, 39, 40, 43, 45, 51, 52, 54, 55, 56, 58, 59 and 66). Positive PCR products (approximately 150 bp) visualized under UV light after electrophoresis in a 1% agarose gel in TBE buffer (0.1 mol/L Tris, 0.09 mol/L Borate, 1 mol/L EDTA) stained with ethidium bromide (0,5 μl/ml) were further analyzed using type-specific primers targeting the E6 region of the HPV genome of HPV types 6,11,16,18,31 and 33 according to a previously published protocol[13]. DNA extracted from HeLa cells infected with HPV18 was used as positive control in the type-specific HPV PCR assays. The PCR procedure was repeated twice for every sample. A representative number of PCR products from each PCR assay was sequenced (ABI PRISM^® ^3730XL DNA Analyzers by Lark Technologies, Inc, UK) and the PCR products were confirmed.

#### Statistical methods

Sensitivity, specificity, positive and negative predictive values were calculated for cytological results and molecular HPV testing against the histological diagnosis of CIN2 and above or CIN3 and above. More specifically, we calculated values considering all cytology results ≥ ASCUS as positive, or all ≥ LSIL as positive or all ≥ HSIL as positive separately. For HC2 we calculated values for any positive result and for positive high risk results (all high risk infections independent of whether they were high risk only or mixed low-high risk infections). For PCR we used either any positive result or positive results for HPV types 16, 18, 31 and 33 only. Positive and negative likelihood ratios were also calculated [14]: likelihood ratios enable the comparison of diagnostic values of tests in a way independent of the prevalence of the disease. The PLR [sensitivity/(1 - specificity)] corrects the true positive rate by the false positive rate of a test while on the other hand NLR [(1 - sensitivity)/specificity] compares the probability of a negative test in persons with disease, compared to the probability of a negative test in persons without disease [14]. Finally, a Cohen's kappa value was calculated for evaluating the agreement between the test results of HC2 and PCR methods.

## Results

### Cytological results

During the 4 year period 1270 women were evaluated (mean age: 34.2 ± 12.1 years old). Table 1 depicts age associations with cytological findings and HPV detection. The vast majority (n = 991, 78%) of the population examined were between 21 and 45 years old. Overall 241/1270 (19%) women were diagnosed with abnormal cytology (Table 1): more specifically, 101 (8%) women exhibited ASC-US, 119 (9.4%) were diagnosed with LSIL, 10 (0.8%) with HSIL, 10 (0.8%) with squamous cell carcinoma and 1 (0.8%) with adenocarcinoma. HSIL or infiltrating carcinoma was the cytological diagnosis in 21/1270 (1.7%) samples. Reporting rates of ASC-US/LSIL combined were 30.6% for women under 21 yrs old, 17.8% for the 22-30 yrs group, 17.7% for 30-45 yrs group, and then declined in older age groups (Table 1). HSIL+ rates increased with age, with 17/21 (81%) HSIL+ lesions diagnosed in women older than 30 years. Seven of the 11 (63.6%) invasive cancer cases were noted after 45 years of age (Table 1). Regarding HPV testing, PCR+ rates were approximately double those of ASC-US/LSIL combined for all age groups up until 65 years (Table 1).

**Table 1:** Age associations with cytological findings and HPV isolation by PCR. Rates are given per total n of women per age group

Age, yrs	HPV PCR data, n (%)			Cytological data, n (%)						
	***HPV (-)***	***HPV (+)***	***Total***	***WNL***	***ASCUS***	***LSIL***	***HgSIL***	***SCC***	***AdenoCa***	**Total**

≤ 21	26(41.9)	36 (58.1)	62	43(69.4)	10 (16.1)	9(14.5)	0(0)	0(0)	0(0)	62

22 - 30	353(67.6)	169 (32.4)	522	425(81.4)	34(6.5)	59(11.3)	3(0.6)	1(0.2)	0(0)	522

30 - 45	304(64.8)	165 (35.2)	469	379(80.8)	43(9.2)	40(8.5)	4(0.9)	3(0.6)	0(0)	469

45 - 65	141(75.4)	46 (24.6)	187	156(83.4)	14(5)	11(5.9)	3(1.6)	2(1.1)	1(0.5)	187

> 65	24(80)	6 (20)	30	26(86.7)	0(0)	0(0)	0(0)	4(13.3)	0(0)	30

**Total**	873	397	1270	1029	101	119	10	10	1	1270

ThinPrep diagnosis in relation to HPV DNA detection by HC2 or PCR testing is depicted in Table 2. HPV was detected by HC2 in 260/1270 (20.4%) samples and by PCR in 397/1270 (31.3%) samples. The two tests gave highly concordant results (Cohen's k value 0.691, 95% CI: 0.664 - 0.718). Discrepancies between HC2 and PCR were observed in 153 specimens. HC2 detected 8 specimens as being positive for HPV that were not detected by PCR (4 with ASCUS-all low risk HPV types; 4 with LSIL, 3 low and 1 high risk HPV types). PCR detected HPV in 145 specimens not detected by HC2, (80 with normal cytology, 45 with ASCUS, 16 with LSIL, 2 with HSIL, 1 with squamous cell carcinoma and 1 with adenocarcinoma). HC2 detected HPV in 17/21 (81%) while PCR detected HPV in 21/21 (100%) samples with HSIL+ cytology (Table 2). In the squamous cell carcinoma and adenocarcinoma categories 2/11 (18.2%) samples tested negative for HPV DNA with HC2, whereas none of these samples was negative with PCR (Table 2). In 21 cases HPV was not detected by either method: eleven cases with ASCUS and ten cases with LSIL.

**Table 2:** ThinPrep diagnosis in relation to HPV DNA detection by HC2 or PCR

HC2	*WNL*	*ASCUS*	*LSIL*	*HSIL*	*SCC*	*AdenoCa*	Total
*HC2 +*	105	45	93	8	9	0	260

*Negative*	924	56	26	2	1	1	1010

**Total**	1029	101	119	10	10	1	1270

							

**PCR**	***WNL***	***ASCUS***	***LSIL***	***HSIL***	***SCC***	***AdenoCa***	**Total**

*HPV X*	159	38	30	0	0	0	227

*HPV 6*	10	4	9	1	0	0	24

*HPV 11*	0	13	13	1	0	0	27

*HPV 16*	0	2§	3	§	5§	0	10

*HPV 18*	0	8§	5	§	1§	1	15

*HPV 31*	1	1§	2	§	§	0	4

*HPV 33*	10	§	§	§	§	0	10

*Multiple HPV types*§	5	20	43§	8§	4§	0	80

*PCR +*	185	86	105	10	10	1	397

*Negative*	844	15	14	0	0	0	873

**Total**	1029	101	119	10	10	1	1270

Abbreviations: HC2: Hybrid Capture 2; HPV: Human Papillomavirus; WNL: Within Normal Limits; Hyperk: Hyperkeratosis; ASCUS: Atypical Squamous Cells of Undetermined Significance; LSIL: Low-grade Squamous Intraepithelial Lesion;

HSIL: High-grade Squamous Intraepithelial Lesion; SCC: Squamous Cell Carcinoma; AdenoCa: Adenococarcinoma

§Denotes the presence of mixed infections that are summarized under multiple HPV types. Nine HPV 16 mixed infections had LSIL, six had HSIL and four SCC. Twenty-seven HPV 18 mixed infections had LSIL, five had HSIL and three SCC. Nine HPV 31 mixed infections had LSIL, one had HSIL and two SCC. Seven HPV 33 mixed infections had LSIL, two had HSIL and three SCC.

HC2 detected infection with high risk or mixed low-high risk HPV types in 40/101(39.6%) of ASCUS cases. PCR detected HPV 16, 18, 31, or 33 (single or multiple infections) in 31/86 (36%) of ASCUS cases. For LSIL the corresponding figures were 77/119 (64.7%) and 63/105(60%) for HC2 and PCR respectively; for HSIL 6/10 (60%) by HC2 and 8/10 (80%) by PCR (Table 2). All cytological diagnoses (10/10) of squamous cell carcinomas tested positive for at least one of the high risk oncogenic HPV types 16, 18, 31, or 33 by PCR (Table 2).

### Histological results

The comparison of ThinPrep diagnoses and histological results is presented at Table 3. In total, 426 women underwent biopsy (Table 3). From patients with ASCUS 58 out of 101 (57.4%) had CIN 1 in biopsy, whereas only 3 (3%) patients had CIN 2 or higher-grade lesions (Table 3). Only two of 119 (1.7%) patients with LSIL had CIN 2. All (100%) cases classified as HSIL with ThinPrep cytology had a biopsy diagnosis of either CIN2 or CIN 3. ThinPrep cytology correctly identified the single case with cervical adenocarcinoma as well as 10 of 11 (90.9%) subjects with a histological diagnosis of SCC (Table 3). One patient diagnosed cytologically as ASCUS had a SCC on subsequent biopsy.

**Table 3:** Thin prep and HPV(+) testing by HC2 or PCR versus biopsy

	Biopsy results						
	**WNL**	***CIN 1*¶**	***CIN 2***	***CIN 3***	***SCC***	***AdenoCa***	

***ThinPrep***							***Total***

***WNL (HPV pos) #***	181	4	0	0	0	0	185

***ASCUS***	40	58	2	0	1	0	101

***LSIL***	0	117	2	0	0	0	119

***HSIL***	0	0	7	3	0	0	10

***SCC***	0	0	0	0	10	0	10

***AdenoCa***	0	0	0	0	0	1	1

***Total***	***221***	***179***	***11***	***3***	***11***	***1***	***426#***

							

***HPV test-HC2***	WNL	*CIN 1*¶	*CIN 2*	*CIN 3*	*SCC*	*AdenoCa*	**Total**

***HC2 +***	104	137	8	2	9	0	260

***Negative ***	959	*44*	*3*	*1*	*2*	*1*	1010

**Total**	1063	*181*	*11*	*3*	*11*	*1*	1270

							

***(+) HPV test-PCR***	WNL	*CIN 1*	*CIN 2*	*CIN 3*	*SCC*	*AdenoCa*	**Total**

**HPV X**	180	46	1	0	0	0	227

**HPV 6**	10	12	2	0	0	0	24

**HPV 11**	1	24	2	0	0	0	27

**HPV 16**	0	4	(3*)	(3*)	6 (4*)	0	10

**HPV 18**	0	13	(6*)	0	1 (3*)	1	15

**HPV 31**	0	4	(1*)	(1*)	(2*)	0	4

**HPV 33**	10	0	(1*)	(1*)	(3*)	0	10

**Multiple HPV types**	7	60	6	3	4	0	80

**PCR (-)**	855	18	0	0	0	0	873

***Total ***	***1063***	***181***	***11***	***3***	***11***	***1***	***1270***

# Data on 844 women with Thin test within normal limits and no HPV detection or colposcopic lesion that did not undergo biopsy are not shown.

¶*2 *Two women had a colposcopic lesion that on biopsy was CIN 1, with normal pap and no HPV

* Denotes the presence of mixed infections that are summarized under multiple HPV types. All multiple infections associated with > = CIN2 had either HPV 16, 18, 31, 33.

Nineteen out of 26 cases (73.1%) with a biopsy diagnosis of CIN 2+ had a HC2 positive test for high risk viruses, whereas PCR detected HPV types 16, 18, 31, or 33 in 21/26 (80.8%) such cases (Table 3). For CIN 3 and invasive carcinoma, HC2 was positive in 11/15 (73.3%), whereas high risk HPV types by PCR were detected in 15/15 cases (100%) (Table 3). Seven of the 11 (63.6%) squamous cell carcinoma cases confirmed by biopsy had a single infection with either HPV type 16 or 18. The single adenocarcinoma case had an infection with HPV type 18 only. Four mixed infections (all had HPV 16, three HPV 18 or HPV 33 and two HPV 31) were diagnosed by biopsy as SCC. Sensitivities, specificities, positive and negative predictive values and positive and negative likelihood ratios for cytological and molecular HPV testing and histological diagnosis of CIN2+ or CIN3+ are provided in Table 4. Positive likelihood ratios (PLR) of 9.4, 3.8 and 3.4 were noted for ≥ LSIL, positive HC2 or positive HPV testing respectively and CIN2+ histology (Table 4). For CIN2+ histological lesion the best positive likelihood ratios (PLR) were observed with Thin prep testing exhibiting ≥ LSIL (PLR = 9.4), or ≥ HSIL (PLR = infinite), and for high risk type of virus detection either by HC2 (PLR = 5.5), or PCR (PLR = 11.4). Similar figures were observed for CIN3 (Table 4). Negative likelihood ratios of 0.13, 0.21, and 0 were noted for cytology ≥ LSIL, positive HC2 or positive HPV testing respectively and CIN2+ histology (Table 4).

**Table 4:** Sensitivity, specificity, positive and negative predictive values and likelihood ratios of the various methods

	No. positive	Sens ≥ CIN2	Spec ≥ CIN2	PPV ≥ CIN 2	NPV ≥ CIN 2	PLR ≥ CIN 2	NLR ≥ CIN2
**ThinPrep Pap** **≥ ASCUS**	241/1270 18.9%	26/26 100%	1029/1244 82.7%	26/241 10.8%	1029/1029 100%	5.8	0

**ThinPrep Pap** **≥ LSIL**	140/1270 11%	23/26 88.5%	1127/1244 90.6%	23/140 16.4%	1127/1130 99.7%	9.4	0.13

**ThinPrep Pap** **≥ HSIL**	21/1270 1.7%	21/26 80.8%	1244/1244 100%	21/21 100%	1244/1249 99.6%	∞	0.19

**HC2 (+) ALL**	260/1270 20.4%	19/26 73.1%	1003/1244 80.6%	19/260 7.3%	1003/1010 99.3%	3.8	0.21

**HC2 (+)** **HR only**	185/1270 14.6%	19/26 73.1%	1078/1244 86.7%	19/185 10.3%	1078/1085 99.4%	5.5	0.31

**HPV PCR (+)**	397/1270 31.3%	26/26 100%	873/1244 70.2%	26/397 6.5%	873/873 100%	3.4	0

**HPV PCR (+)** **16/18/31/33**	109/1270 8.6%	21/26 80.8%	1156/1244 92.9%	21/109 19.3%	1156/1161 99.6%	11.4	0.21

	**No. positive**	**Sens** **≥ CIN3**	**Spec** **≥ CIN 3**	**PPV** **≥ CIN 3**	**NPV** **≥ CIN 3**	**PLR** **≥ CIN 3**	**NLR** **≥ CIN 3**

**ThinPrep Pap** **≥ ASCUS**	241/1270 18.9%	15/15 100%	1029/1255 82%	15/241 6.2%	1029/1029 100%	5.6	0

**ThinPrep Pap** **≥ LSIL**	140/1270 11%	14/15 93.3%	1129/1255 90%	14/140 10%	1129/1130 99.9%	9.3	0.07

**ThinPrep Pap** **≥ HSIL**	21/1270 1.7%	14/15 93.3%	1248/1255 99.44	14/21 66.7	1248/1249 99.9%	166.6	0.067

**HC2 (+) ALL**	260/1270 20.4%	11/15 73.3%	1006/1255 80.2%	11/260 4.2%	1006/1010 99.6%	3.7	0.33

**HC2 (+)** **HR only**	185/1270 14.6%	11/15 73.3%	1081/1255 86.1%	11/185 5.9%	1081/1085 99.6%	5.3	0.31

**HPV PCR (+)**	397/1270 31.3%	15/15 100%	873/1255 69.6%	15/397 3.8%	873/873 100%	3.2	0

**HPV PCR (+)** **16/18/31/33**	109/1270 8.6%	15/15 100%	1161/1255 92.5%	15/109 13.8%	1161/1161 100%	13.4	0

*HSIL*: High-grade squamous intraepithelial lesion, *Sens*: sensitivity, *Spec*: specificity, *PPV*: Positive predictive value, *NPV*: Negative predictive value, *PLR*: positive likelihood ratio, *NLR*: negative likelihood ratio

## Discussion

This study depicts the successful use of both HC2 and PCR in liquid based ThinPrep samples. Use of the same samples for both DNA tests was done to avoid bias. The HC2 kit assesses the presence of 18 low risk (i.e. 6,11,42,43 and 44) and high risk (i.e. 16, 18, 31, 33, 35, 39, 45, 51, 51, 56, 58, 59 and 68) HPV types [15,16]. On the other hand, using the L1 consensus primers GP5^+^/GP6^+ ^by PCR, we were able to screen for 22 HPV types. The concordance rate between the PCR and HC2 test results was high in our series. Both molecular tests exhibited high reproducibility measured by Cohen's kappa value of 0.691, similar to previous reported results [17,18]. Although not all studies use the same PCR protocol, the concordance between HC2 and PCR testing across various studies is generally high, usually exceeding 80% [17-20]. It appears that after a certain DNA concentration cutoff point, HC2 sensitivity increases, carrying a certain risk for false positive results[17]. Newer generation kits decrease rates of false positive testing[21]. Certain cutoff levels for relative light units compared to the ones generated by the positive control samples to increase the specificity of the assay without compromising sensitivity may be used[18]. One explanation for some of the discordant results between HC2 and PCR in our study is the fact that GP5+/GP6+ primers may actually miss some cases deleted in the L1 region of the virus. In addition, some of the samples exhibiting discordance may have infection with HPV types such as HPV 42, 44 or 68 not amplified by the used primers, but still detectable by the HC2 cocktails. Moreover, HC2 probe B cocktail can react with phylogenetically related HPV types not represented in the probe such as HPV 67, CP 6108, and CP8061. Finally, the prevalence of latent infection differs across studies, depending upon demographic parameters; this may also affect the sensitivity and specificity of the two tests.

Our results confirm the higher prevalence of HPV infection in women with abnormal cytology, in concordance with most studies so far published, that have observed that the increase in HPV prevalence is related to the increasing grade of squamous intraepithelial lesions [22-26]. Rates of HPV detection in samples with normal cytology vary widely in the literature in women with normal cytology and range from 4.9% to 30.4% for the HC2 assay and from 3 to 34.3% for PCR testing [18,19,21,25,27]. This wide range is explained by the different nature of participating populations in such studies, and by technical evolutions in the diagnostic tests used[21,28]. The higher analytical sensitivity of PCR explains the higher detection rates compared to HC2 in samples with low-grade cytological and no histological abnormalities and may be suggestive of the presence of latent HPV infection requiring a molecular test with higher analytical sensitivity.

Similar to other observations, both hybrid capture and PCR HPV DNA testing showed a strong correlation with the diagnosis of CIN lesions or squamous cell carcinoma [23,29]. The extremely high negative predictive values of both molecular tests underlie their importance in screening for high grade lesions and are in concordance with previous publications[18]. In high grade cytological and histological lesions, HPV detection rates appeared to be a little higher with PCR than with HC2 testing; for example HC2 did not identify high risk viruses in seven instances of CIN2+ histological lesions, in which cases PCR was positive. However positive and negative likelihood ratios were similar for positive HC2 (any result) or PCR - HPV positive testing (any result) both for CIN2+ or for CIN3+ histology. Actually, HC2 had a better PLR while PCR had a better NLR in both histological categories. This means that with a positive HC2 there was a greater likelihood of disease (≥ CIN2) than with PCR, while with a negative HPV PCR there was a lesser likelihood of disease (≥ CIN2) than with HC2. Nevertheless, in essence both tests gave similar results. Likelihood ratios can be used to calculate the odds of post test probabilities if multiplied by the odds of the prevalence of the disease. For example, a patient in our study had a post test probability of disease (CIN2+) of only 5.1% (using the Bayes nomogram) if she had a negative HC2 test (according to our study she had a pre-test probability of 20.4% of having a positive test). Similarly a negative PCR for high risk types 16, 18, 31 and 33 has a post test probability of disease (CIN2+) of 1.93% (Table 4). The limitation of a population that is not systematically screened, but is consecutively enrolled for deriving this data is of course recognized. However, both molecular HPV tests did not outperform cytology that had comparable if not better results, especially if the HSIL+ cytological lesion cutoff point was used. In our study all HSIL lesions by cytology were either CIN 2 or CIN 3 by histology and all cytological diagnoses of invasive carcinomas were subsequently confirmed by histology. Possible explanations for this observation include the following: a) the experience of all doctors involved; b) the increased alertness of both the referring gynecologist and the cytopathologist examining the slides; c) the fact that the Department of Cytopathology is accredited and therefore undergoes continuous quality assessment/quality control evaluation.

Although none of the patients with normal cytology and an HPV infection had a biopsy proven lesion higher than CIN 1 in our study, these women should not be considered false-positive but as having a real risk for progression to abnormal cytological findings and cervical neoplasia [30-33]. It is known that development of precancerous lesions may shortly follow infection with HPV, despite the belief that long-term infection is a prerequisite for such an event [34]. Thus, these women should be prospectively followed by their gynecologist and submitted to cytology and other testing, as appropriate[35,36]. The use of HPV testing has been recommended for women with ASCUS[37] and it has been shown that approximately one third of women with HSIL are subsequently identified from an initial ASCUS diagnosis [25]. These women, after positive HPV DNA testing should be referred for colposcopy[38]; our study underlines this argument, since one woman with ASCUS, had a single infection with HPV type 16 and biopsy disclosed squamous cell carcinoma. Nowadays, in the revised Bethesda 2001 classification system this case would probably be diagnosed as ASC-H. A molecular technique, more sensitive and specific than HC2 may be more appropriate for such patients.

The current observations further confirm the association of high risk HPV types with cytological detection of HSIL or invasive carcinoma but also with histologically confirmed premalignant or malignant lesions [39-41]. All cases of CIN 2, CIN 3 or carcinomas harbored single or multiple HPV high risk oncogenic type infections. In our study single infections with HPV types 16 or 18 in cases of invasive carcinoma were strong evidence for this association and support the strong rationale for preventive HPV vaccination in our population.

The rate of invasive carcinoma discovered in this study is indicative of a largely unscreened population. The reasons and possible solutions for this observation are an important public health issue and should be further investigated. Moreover the effect of the introduction of newer techniques in population screening is a matter of intense research. Despite the excellent results with cytology in this study, it is well known that screening for cytological changes may have limited sensitivity and findings are not always reproducible [8]. In a country where screening is largely opportunistic and based on self-referral results of cytological testing are expected to be much worse than the ones presented here. Moreover, molecular HPV testing should not be introduced without careful planning; results of such testing should be communicated and explained appropriately in the context of prevalence of the disease.

## Conclusion

In conclusion, the present study was more of a technical nature and focused on strengths and weakness of current screening methods in correlation with histological findings. HPV rates were high in high grade lesions in accordance to published literature however HPV was also identified in a large fraction of samples with normal cytology or in samples with normal biopsies. The best approach for such samples will be guided after careful identification of the local epidemiology of the transmission and risk factors for HPV acquisition. Newer methods assessing the integration of the virus may be more appropriate in the workup of such cases. In our study in an referral center with experienced doctors, thin prep cytology and molecular HPV tests performed equally well as screening tests in all scenarios and sometimes were complementary to each other. As newer methods to detect HPV continue to evolve it is important to recognize the clinicopathological correlates of such testing. This will lead to more appropriate screening strategies in the post-vaccine era.

## Competing interests

The authors declare that they have no competing interests.

## Authors' contributions

ST, AC, JG, and PK conceived and participated in the design and coordination of the study; ZV collected thin prep and histology samples; JG, AT and PK performed the thin prep evaluation; AC performed the molecular studies; JP and PK performed the pathological evaluation; ST, JP, AP and PK performed the statistical analysis and analyzed the data; ST, AC, JP and PK drafted the first draft; All authors reviewed and critically revised the first draft; All authors read and approved the final manuscript.

## Pre-publication history

The pre-publication history for this paper can be accessed here:

http://www.biomedcentral.com/1471-2407/10/53/prepub
